# Addressing Challenges for Psychotherapy Supervision in Global Mental Health: Experiential Learnings From Rural Nepal

**DOI:** 10.21203/rs.3.rs-4499074/v1

**Published:** 2024-06-26

**Authors:** Pragya Rimal, Srijana Shrestha, Rekha Khatri, Sabitri Sapkota, Sikhar Bahadur Swar, Madhur Basnet, Kripa Sigdel, Sunita Jirel, Bibhav Acharya

**Affiliations:** Possible; Wheaton College; The University of Sydney; Possible; Bayalpata hospital; B. P. Koirala Institute of Health Sciences; Possible; Charikot hospital; University of California San Francisco

**Keywords:** supervision, psychology, global mental health, LMIC, Nepal, psychotherapy

## Abstract

**Background:**

As the field of global mental health grows, many psychotherapy trainees will work across cultures in low-resource settings in high-income countries or in low- and middle-income countries. Faculty members and mentors may face several challenges in providing supervision for psychologists in low-resource settings. As such, there is a need to develop best practices for psychotherapy supervision in global mental health.

**Methods:**

We describe the common challenges and potential strategies in psychotherapy supervision based on our research, clinical, and academic partnerships between academic institutions, a nonprofit organization, and the Nepali government.

**Results:**

The strategies and considerations we have found helpful include focusing on therapies with strong behavioral and interpersonal (rather than emotional or cognitive) components and using locally validated therapies or standard manuals that have been endorsed by the WHO for low-resource settings. Other strategies include providing psychotherapy training for local psychiatrists who may be in supervisory roles and gaining competence in navigating different expectations of social structures and family dynamics.

**Conclusion:**

Supervisors face many challenges while supporting trainees and early psychologists in global mental health settings. While ensuring local adaptation, key considerations can be developed into best practices to support psychiatrists, supervisors, and trainees based in low- and middle-income countries.

## BACKGROUND

Supervision in psychotherapy is defined as the process of receiving guidance and feedback from a more experienced therapist or supervisor with the goal of reflecting on one’s practice and improving therapeutic skills (1). Supervision is vital for improving the professional competencies (2, 3) of trainees and early career professionals and for improving clients’ clinical outcomes. Depending on the trainees’ needs, supervisors can operate as mentors, teachers, or consultants and help trainees with clinical and ethical practices and professionalism while also providing personal support. Although the protocols and expectations for clinical supervision in psychotherapy are well established in high-resource settings (4), many considerations need to be employed in Global Mental Health (GMH), particularly in low- and middle-income countries (LMICs) and resource-constrained areas in high-income countries (HICs).

GMH is a relatively new discipline focused on improving mental health research and practice around the world, particularly in LMICs and in resource-constrained areas in HICs (5, 6). As the field grows, more trainees work across cultures, nationalities, languages, and, often, different countries. There are many challenges in providing mental healthcare in cross-cultural settings, and consequently, there are unique opportunities for providing supervision. As such, there is a need to provide best practices for psychotherapy supervision in GMH so that trainees can work in low-resource settings to improve mental healthcare while maintaining fidelity to evidence-based therapies and meeting their learning objectives. In this paper, we describe some of the challenges and strategies in supervision based on our experience in rural Nepal.

## METHODS

Study site: The experiences shared are based on our work with a nonprofit Possible in Nepal. Possible works with Nepal-based partners and US-based academic institutions to support rural healthcare delivery systems owned by the government of Nepal and managed by local organizations. In this paper, we share experiences from two rural primary care clinics in Nepal. The clinics are based in Achham and Dolakha, and both study sites have endured humanitarian crises over the last two decades. Achham, located in the far west region, is one of the poorest areas of Nepal and has suffered the consequences of a decade-long internal conflict and concentrated HIV epidemic poverty(6). The second site in Dolakha had 80% of its healthcare infrastructure destroyed by an earthquake in 2015 (7). Since 2015, the authors have collaborated with Nyaya Health Nepal, a local nonprofit, to research and support mental health and behavioral healthcare. This partnership involves training mental health specialists and nonspecialists, including primary care providers, to deliver high-quality mental healthcare (8–10). Together, the clinics serve more than 150,000 outpatient visits annually. The mental health specialists at the study sites included psychologists (with formal training in psychology at the master’s level) and counsellors with three to six months of training to conduct psychosocial evaluations and deliver basic psychotherapy, relaxation techniques, and psychoeducation (11). Nonspecialists include health assistants who have three years of medical training and primary care physicians(9, 12). They are trained in basic principles of psychotherapy, such as improving interpersonal communication skills with patients. In addition, community health workers (salaried and supervised local women with a minimum of grade 10 education) (7, 13) are trained in motivational interviewing to help improve patients’ treatment adherence (16). Funding for this set up comes from the Nepali government, research grants, academic support from universities, and philanthropic support. Our experiences, which we have described in detail in the literature, are based on adapting, implementing, and studying evidence-based mental health services in resource-limited settings(9, 14–17).

## RESULTS

### Challenges and opportunities for using psychiatrists as psychotherapy supervisors in resource-limited settings

As defined by the WHO, when we say “psychologists”, we include graduates who have completed university-level education and who work in mental health with a specialization in clinical psychology (18). Despite the use of this broad definition, there are more psychiatrists than psychologists in LMICs. A WHO report on the mental health workforce estimates that globally, there are 0.9 psychologists, compared to 1.3 psychiatrists per 100,000 people. The ratio is the lowest in the African and Southeast Asian regions, with 0.1 psychologists compared to 0.7 and 0.4 psychiatrists, respectively (18). Because of the small number of psychologists in resource-limited settings, psychiatrists (rather than senior psychologists) are often assigned supervisory roles for psychotherapy trainees, especially those visiting from HICs for global health rotations. However, although psychiatry training includes some psychotherapy training in HICs, this is almost nonexistent in LMICs (19, 20). As such, while psychiatrists can be supervisors for certain aspects of a psychologist’s work (behavioral interview, diagnostic evaluation, and psychoeducation), they are unlikely to be equipped to provide supervision in psychotherapy. An important exception could be made for psychiatrists who have completed fellowship programs in psychotherapy. While such cases may be less common in LMICs, a psychiatrist who has completed a fellowship in addiction psychiatry would likely receive supervised training in motivational interviewing. In addition, psychiatry residency programs may not integrate training in public health, teaching and managerial skills, which increases the difficulties of supervision in GMH (21).

To address this issue, first, academic programs should be aware that psychiatrists’ training in psychotherapy may be limited and not assume that they can provide psychotherapy supervision (22). In our program, we have provided opportunities for psychiatrists to access training in psychotherapy, as this was more viable than building a pipeline for local, senior psychologists. Given the psychiatrists’ background in mental health, we find that if they are motivated to learn therapy, they may require less extensive training. Psychiatrists in our program have gained basic competencies after attending short 2–3-day courses on Cognitive Behavioral Therapy (CBT), Psychological First Aid (PFA), and problem-solving therapy (PST). If such training is not feasible, the supervising faculty in the academic centers may need to deliver “train the supervisor” training for psychiatrists (23). If long-term partnership and funding are feasible, this can be combined with parallel investments in developing psychology training programs to establish a pipeline for local senior psychologists.

### Mismatch in common psychotherapeutic modalities used in high- and low-income settings

In GMH, psychotherapy trainees may work in LMICs or resource-constrained settings in HICs. However, their primary faculty supervisor is often a psychologist based in an academic setting in an HIC. As such, HIC-based supervisors may not be fully aware of therapies that are locally available and/or validated. Many academic settings in HICs employ training in therapies such as CBT and psychodynamic psychotherapy (24–26). Without cross-cultural adaptation, there is very little evidence for the use of these therapies in LMICs (27, 28). In such cases, HIC-based supervisors may need to familiarize themselves with modalities that have evidence in LMICs and work in close collaboration with local clinicians.

A widely used technique in LMICs is Problem Management Plus (PM+), which is derived from PST and acknowledges that not all problems can be “solved” but can likely be managed (29). Similarly, another modality with evidence in LMICs is the PFA, which is used after a crisis vent (30–32)

The thinking healthy programme adapted from CBT for perinatal depression has been found to be effective in multiple LMICs (33–36). Due to a greater emphasis on interpersonal relationships than on intrapersonal psychological processes, group interpersonal therapy (IPT) is particularly relevant in such settings (37). In addition, mental health literacy among patients may be quite low, and sessions that focus on the introduction of therapy (e.g., explaining the relationships among emotions, thoughts, and behaviors) may take longer than usual. A helpful resource for introducing clients to the ideas of mind and psychotherapeutic approaches is the WHO’s “Doing What Matters in Times of Stress,” which is an illustrated guide that walks clients through the basics of mental health self-management skills (38). The WHO maintains a repository and standard manuals for these therapies. HIC-based supervisors will need to become familiar with these techniques and pay particular attention to modifications/adaptations and other differences between how these therapies are delivered in high- vs low-resource settings.

A related challenge is the limitation in directly observing client-provider interaction to provide feedback to the provider. Given that many sites may be rural and difficult to access, it is often challenging to ensure that supervisors can directly observe such interactions, which is a fundamental aspect of psychotherapy supervision. Our team recently completed two studies that leverages digital tools to address this challenge: with the client’s informed consent, we audiotaped client-provider interactions, which could then be reviewed by specialists to provide feedback to the provider (39, 40).

### Local culture, context, idioms of distress, and mechanisms of change

Evidence suggests that in settings where mental health is stigmatized and there is a lack of access to effective mental health treatment, people are more likely to express distress through physical problems rather than through the use of emotional language (41–43). This is more likely to happen in government-funded settings that serve even more marginalized populations within low-resource settings who have had little exposure to effective mental health treatment and interventions that destigmatize mental illness. In such settings, distress may be primarily described in physical (“I have tingling sensations all over”), behavioral (“She spends too much time just staring”), and interpersonal terms (“My son does not like to socialize and doesn’t listen to me”) rather than emotional terms (“I feel like I am depressed”) or intrapersonal terms (“I am worried about my future”). The mechanism of improvement may also be primarily behavioral rather than emotional or cognitive (e.g., behavioral activation may be more acceptable and effective, both for the client and their family members, than completing thought records or exploring the source of emotions). While there will certainly be exceptions, both trainees and supervisors need to be aware of these potential differences. If problems are expressed behaviorally, therapies such as PST and interpersonal psychotherapy could be more relevant than therapies that are largely focused on emotions. Although we do not recommend completely avoiding any discussions of emotions, we have found that uptake is greater for modalities that are primarily interpersonal and behavioral. Another important consideration is that given the potentially limited access to physical health services, supervisors need to be aware that patients may not have received a thorough medical rule-out, and consequently, the presence of somatic symptoms may indicate a referral to a primary care provider.

### Involvement of family members during therapy

In many HICs, psychotherapy places a high emphasis on maintaining client confidentiality and autonomy and occurs one-on-one. In contrast, in many resource-limited settings such as Nepal, it is the norm for family members to accompany clients to the clinic and attend clinical visits, including therapy sessions. Several studies have demonstrated that involving family members can help families understand mental health problems, support the client, promote treatment adherence and help clients reach therapeutic goals (27, 44). When trainees and their supervisors insist on individual sessions, clients may respond with suspicion or nonadherence. While the decision to involve family members should be at the client’s discretion, trainees need support in managing such situations to attend to the client, listen to the accompanying family, and address family dynamics.

Supervisors with experience in family-focused and group therapy will be able to assist trainees in navigating sessions that may have traditionally been individual-focused. The presence of family members can be leveraged to improve the client’s situation (e.g., developing a behavioral activation plan in consultation with family members and recruiting them to overcome anhedonia in the client). Moreover, it remains important to acknowledge that family members may sometimes contribute to the client’s distress, and trainees will also need support in navigating such situations by connecting the client to social/legal services.

### Providing longitudinal care in resource-limited settings

Clients in LMICs may often face challenges in engaging in long-term weekly visits due to barriers such as the need for extended travel time to seek care (in our setting, the average length of travel to the nearest health facility is 2–3 hours each way), lack of a reliable mode of transportation, and competing demands (e.g., prohibitive opportunity costs for daily wage earners or people with caregiver responsibilities). While telecommunication and remote care can help alleviate some of these barriers, they may not be feasible due to inadequate digital infrastructure.

To account for these barriers, trainees should be encouraged to deliver brief therapies such as psychoeducation that focus on empowerment and skills building (45). Psychoeducation combines CBT, group therapy and education to provide focused information about mental illness, problem solving, communication and assertiveness training to help clients understand mental health treatment and coping strategies (45–47). Recognizing the challenges in longitudinal care, supervisors can support trainees by ensuring that they address the core components of illness in the first single session, knowing that the client may not be back for a follow-up session at all. Single session therapy is often deprioritized in academic settings but can be effective in treating common mental health problems (48, 49). We suggest treating the first visit as a single session of therapy and providing clients with information and a plan of action to address their immediate mental health needs (50–53). Multisession therapy should be attempted after it has been determined that the client can return for subsequent therapy sessions.

## DISCUSSION

Our experiences are based on a partnership between academic institutions, nonprofit primary care clinics in rural Nepal and the Nepali government. Although some suggestions we propose (e.g., providing access to training to psychiatrists or psychologists with experience in supervision) may still be unfeasible due to a lack of experts in other GMH settings, the common challenges and potential solutions that we share may be generalized to other LMICs and to resource-constrained areas in HICs, including work with refugee, immigrant, and indigenous populations.

## CONCLUSIONS

To the best of our knowledge, there are no guidelines or best practices for providing psychotherapy supervision in GMH settings. The common challenges and potential solutions that we share may be generalized to other LMICs and to resource-constrained areas in HICs and minority populations.

Both trainees and supervisors should be aware of the cultural and contextual differences in the understanding of mental health issues in the communities where they work. Utilizing the various strategies and considerations noted above, trainees and supervisors can develop best practices to provide effective psychotherapy supervision in low-resource settings globally.

## Data Availability

Data sharing not applicable to this article as no datasets were generated or analyzed during the current study.

## Abbreviations

GMH: Global Mental Health
HICs: High-income countries
LMICs: Low- and middle-income countries
CBT: Cognitive Behavioral Therapy
PFA: Psychological First Aid
PM+: Problem Management Plus
PST: Problem-solving therapy
IPT: Interpersonal therapy
